# Knowledge on intrapartum care practices among skilled birth attendants in Cambodia—a cross-sectional study

**DOI:** 10.1186/s12978-021-01166-z

**Published:** 2021-06-09

**Authors:** Mitsuaki Matsui, Yuko Saito, Rithy Po, Bunsreng Taing, Chamnan Nhek, Rathavy Tung, Yoko Masaki, Azusa Iwamoto

**Affiliations:** 1 Department of Global Health, Nagasaki University School of Tropical Medicine and Global Health, Sakamoto 1-12-4, Nagasaki, 852-8523 Japan; 2 Phnom Penh Municipal Health Department, Ministry of Health, Street 2011, Sangkat Phnom Penh Thmey, Khan Sensok, Phnom Penh, Cambodia; 3 Project for Improving Continuum of Care with focus on Intrapartum and Neonatal Care in Cambodia, Japan International Cooperation Agency, Building 61-64, Preah Norodom Blvd, Phnom Penh, Cambodia; 4 Kampong Cham Provincial Health Department, Ministry of Health, Preah Kosamak Nearyroth, Kampong Cham, Cambodia; 5 Svay Rieng Provincial Health Department, Ministry of Health, National Road #1, Svay Rieng, Cambodia; 6 National Maternal and Child Health Center, France Street, Sangkat Srah Chork, Khan Daun Penh, Phnom Penh, Cambodia; 7 Bureau of International Health Cooperation, National Center for Global Health and Medicine, Toyama 1-21-1, Shinjuku-ku, Tokyo, 162-8655 Japan

**Keywords:** Skilled birth attendant, Midwifery (MeSH terms), Intrapartum care, Cambodia (MeSH terms), Partograph

## Abstract

**Background:**

Delivery is a critical moment for pregnant women and babies, and careful monitoring is essential throughout the delivery process. The partograph is a useful tool for monitoring and assessing labour progress as well as maternal and foetal conditions; however, it is often used inaccurately or inappropriately. A gap between practices and evidence-based guidelines has been reported in Cambodia, perhaps due to a lack of evidence-based knowledge in maternity care. This study aims to address to what extent skilled birth attendants in the first-line health services in Cambodia have knowledge on the management of normal delivery, and what factors are associated with their level of knowledge.

**Methods:**

Midwives and nurses were recruited working in maternity in first-line public health facilities in Phnom Penh municipality, Kampong Cham and Svay Rieng provinces. Two self-administered questionnaires were applied. The first consisted of three sections with questions on monitoring aspects of the partograph: progress of labour, foetal, and maternal conditions. The second consisted of questions on diagnostic criteria, normal ranges, and standard intervals of monitoring during labour. A multiple linear regression analysis was performed to identify relationships between characteristics of the participants and the questionnaire scores.

**Results:**

Of 542 eligible midwives and nurses, 523 (96%) participated. The overall mean score was 58%. Only 3% got scores of more than 90%. Multivariate analysis revealed that ‘Kampong Cham province’, ‘younger age’, and ‘higher qualification’ were significantly associated with higher scores. Previous training experience was not associated with the score. Substantial proportions of misclassification of monitoring items during labour were found; for example, 61% answered uterine contraction as a foetal condition, and 44% answered foetal head descent and 26% answered foetal heart rate as a maternal condition.

**Conclusion:**

This study found that knowledge was low on delivery management among skilled birth attendants. Previous training experience did not influence the knowledge level. A lack of understanding of physiology and anatomy was implied. Further experimental approaches should be attempted to improve the knowledge and quality of maternity services in Cambodia.

**Supplementary Information:**

The online version contains supplementary material available at 10.1186/s12978-021-01166-z.

## Plain language summary

Pregnancy and childbirth are natural phenomena, but sometimes have risk for mothers and babies. Therefore, childbirth should be carefully and continuously monitored by the health care professional. The ‘partograph’ is a useful tool that defines three monitoring aspects of the delivery progress, and conditions of the mother and intrauterine baby. However, it is often used inaccurately or inappropriately in low- and middle-income countries. We hypothesised that health professionals who assist childbirth cannot effectively monitor delivery conditions because their knowledge is insufficient. Therefore, we evaluated the knowledge on monitoring the process of childbirth and explored factors which affect the level of knowledge among health care providers in Cambodia.

Midwives and nurses were targeted in this study who deal with normal deliveries in the capital city and two provinces. The questionnaire was designed to evaluate if their knowledge on three monitoring aspects is accurate.

Of 542 eligible personnel, 523 (96%) participated. The mean score was 58%. Only 3% got scores of more than 90%. According to the statistical analysis, ‘working in Kampong Cham province’, ‘younger age’, and ‘higher qualification’ were significantly associated with higher scores. Previous training experience was not associated with the score.

This study found that basic knowledge was low on delivery management among health care providers. We suspect that a deficiency of basic medical knowledge, such as physiology and anatomy, causes the lack of knowledge on the childbirth process. Further intervention should be attempted to improve the knowledge and quality of maternity services in Cambodia.

**Supplementary Information:**

The online version contains supplementary material available at 10.1186/s12978-021-01166-z.

## Introduction

The process of delivery and childbirth is characterised by dynamic changes in anatomy and physiology for mother and foetus. As delivery progresses, uterine contractions become intense and the foetus in utero is exposed to extremely low levels of oxygen [1]. Asphyxia is the most common cause of neonatal deaths in south Asia and sub-Saharan Africa [2]. Delivery is also a critical moment for pregnant women, and it has been estimated that 28% of maternal deaths in the world occur during the intrapartum or immediate postpartum period [3]. Another estimate is that 40–45% of maternal deaths, stillbirths, and neonatal deaths occur during or within 24 h after delivery in south Asia and sub-Saharan Africa [2]. Therefore, to ensure a normal birth process careful monitoring is essential throughout the delivery procedure so that abnormal signs are detected early.

The partograph is a useful tool for monitoring and assessing labour progress as well as maternal and foetal conditions [4]. It defines the key items to be monitored, their normal ranges, and the standard monitoring intervals. Although the latest recommendations from WHO emphasise to not follow the cervical dilatation rate threshold of 1 cm per hour during the active phase in the first stage of labour [5], the usefulness of the partograph is not hampered. It is still necessary to plot cervical dilatation versus time on the cervicograph to confirm the progress of labour. Therefore, the utilisation of the partograph helps to identify labour dystocia and risks for adverse birth outcomes [6]. However, it has been shown that the partograph is often used inaccurately or inappropriately, particularly in low- and middle-income countries [7–11].

Cambodia successfully decreased maternal mortality to the target set in the Millennium Development Goals (MDGs) [12]. The latest Cambodia Demographic and Health Survey in 2014 has shown that the maternal mortality ratio and the proportion of deliveries attended by skilled health professionals were 170 per 100,000 live births and 89%, respectively [13]. The Cambodian Ministry of Health has built on the MDGs success and has placed maternal mortality reduction at the top of its third Strategic Health Plan 2016–2020 [14]. Improving the quality of health services is a key strategy for reduction and evidence-based practice is a major element of quality of care. This initiative corresponds with the desire to increase the competency of skilled birth attendants (SBA), which was recently redefined jointly by WHO, UNFPA, UNICEF, ICM, ICN, FIGO, and IPA. It includes: (i) to provide and promote evidence-based, human-rights-based, quality, socio-culturally sensitive, and dignified care to women and newborns; (ii) to facilitate physiological processes during labour and delivery to ensure a clean and positive childbirth experience; and (iii) to identify and manage or refer women and/or newborns with complications [15]. Competency is measured by performance, which is based on the knowledge, skills, and behaviours required as a professional.

Studies in Cambodia revealed that there was a gap between practices and evidence-based guidelines, although substantial technical support in obstetric and midwifery training courses had been provided for two decades [16–20]. One possible reason behind this gap is a lack of evidence-based knowledge in maternity care [16, 20]. However, there has been no systematic assessment of knowledge on intrapartum care practices among skilled birth attendants in Cambodia.

## Methods

### Study aims

This study aimed to address two questions: (i) to what extent skilled birth attendants in the first-line health services in Cambodia have sufficient knowledge on management of intrapartum care; and (ii) what factors are associated with the level of knowledge, especially previous training experiences among the health care providers.

### Study design

This study employed a cross-sectional design.

### Study setting

This study was jointly organised by a research project on the impact of 'evidence-based midwifery care' on maternal and neonatal outcomes, and a bilateral technical cooperation project between the Cambodian and Japanese governments. The former was conducted in selected first-line public health facilities in the capital city Phnom Penh. The latter is implemented by the National Maternal and Child Health Centre (NMCHC) in Cambodia and Japan International Cooperation Agency (JICA), called ‘Project for Improving Continuum of Care with focus on Intrapartum and Neonatal Care in Cambodia’ (IINeoC). Its target areas are Kampong Cham and Svay Rieng provinces, which are in the central lowlands of the Mekong river and in southeastern Cambodia next to Vietnam, respectively. This study was conducted as a baseline survey in the two projects.

### Target facility and population

Midwives and nurses were recruited working in maternity in the first-line public health facilities in Kampong Cham and Svay Rieng provinces, and Phnom Penh municipality. Health facilities which had 20 deliveries or more per month were selected for Phnom Penh, whereas all facilities were involved in Kampong Cham and Svay Rieng provinces. A list of health care personnel assisting deliveries was prepared prior to the survey. All participants were invited to the municipal or provincial health departments. The questionnaires were applied to those who agreed to participate. Data collection was conducted between 11 and 15 June 2018 in Svay Rieng, 9 and 12 July 2018 in Kampong Cham, and 16 and 17 July 2018 in Phnom Penh.

### Measurement of knowledge

Two sets of self-administered questionnaires were prepared to measure knowledge on intrapartum care. The first questionnaire consisted of three sections with questions on monitoring items for three aspects of the partograph: progress of labour, foetal condition, and maternal condition. Every question was open-ended; therefore, the participants were asked to conduct free listing of the items. The answers were scored, and one point was given for each correct item specified by a participant. There are ten correct items in the first questionnaire, and therefore the total score ranges between zero and ten. After completion of the first questionnaire, the second one was distributed. It has sixteen questions which consist of nine items on knowledge of criteria of diagnosis or the normal range of diagnostic parameters, and seven questions on standard interval of monitoring during labour. Each question in the second questionnaire had a single correct answer except for questions on diagnosis of hypertension. The questions on hypertension displayed 42 different results of blood pressure and the participant was asked to distinguish instances of hypertension. A score of 0.024 was given for each of 42 correct answers. One point was given to each correct answer in the other questions. All twenty-six components of the questionnaire with the expected answers are shown in Table 1.

**Table 1:** Contents of the questionnaires to measure knowledge on intrapartum care

I. Knowledge on monitoring items (10 items)	
Category	Expected answer
Progress of labour	Cervical dilatation
	Uterine contraction
	Foetal head descent
Foetal condition	Foetal heart rate (FHR)
	Amniotic fluid
	Moulding of foetal head
Maternal condition	Pulse
	Blood pressure
	Body temperature
	Urine volume

II-1. Knowledge on criteria for diagnosis or normal range of the items (9 items)		
Category	Question	Expected answer
Progress of labour	Beginning of active phase in the 1st stage	From 3 cm of dilatation of uterine cervix
	End of 1st stage; or beginning of 2nd stage	At full dilatation of uterine cervix
	End of 2nd stage; or beginning of 3rd stage	At expulsion of baby
	End 3rd stage; or beginning of 4th stage	At expulsion of placenta
	End of 4th stage	2 h after delivery
Foetal condition	Upper normal limit of FHR	160 beats per minutes
	Lower normal limit of FHR	120 beats per minutes
Maternal condition	Diagnosis of hypertension	Systolic blood pressure 140 mm Hg or more; or Diastolic blood pressure 90 mm Hg or more
	Abnormal amount of PPH	500 ml or more

II-2. Knowledge on recommended interval of monitoring (7 items)		
Category	Question	Expected answer
Progress of labour	Cervical dilatation, 1st stage	Every 4 h
	Uterine contraction, 1st stage	Every 30 min
Foetal condition	FHR, 1st stage	Every 30 min
	FHR, 2nd stage	Every 5 min
Maternal condition	Pulse	Every 1 h
	Blood pressure	Every 1 h

The most recent national protocol was referred, Safe Motherhood Clinical Management Protocols for Health Centers (2016) [21]. A draft of the questionnaire was prepared by the researchers (MM, YS, YM, and AI), and to make a final version it was consulted in a meeting involving obstetrics and maternity care experts from the Cambodian Society of Gynaecology and Obstetrics, the Cambodian Midwives Association, and NMCHC. The questionnaires were made in the Cambodian language (Khmer), which was confirmed by backtranslation into English. All technical terms were taken from the protocol.

### Information on study participants

Information was collected on the participants regarding age, qualification, number of deliveries assisted in the prior month, and previous training experience. We selected three training courses as potential contributing factors on knowledge improvement, because these include a topic of monitoring and evaluation during labour. The titles of the courses are ‘Health centre midwifery course in NMCHC (HC-MW)’, ‘Partograph’, and ‘Basic Emergency Obstetric and Neonatal Care (BEmONC).’ The details of the training are described in Annex 1 (as an Additional file 1).

### Data processing and analysis

The sum points for each participant were calculated. Univariate analyses by Student’s t-test or analysis of variance, and a multiple linear regression analysis were performed to identify the relationships between the characteristics of the participants and their received score, using Stata version 15 (Stata Corp, USA).

## Results

### Participants and their characteristics

Of 542 eligible health care personnel in the three regions, 523 (96%) from 157 health facilities participated. Characteristics of the participants are shown in Table 2. Distribution of age was bimodal with modes in the twenties and forties. Midwife was dominant as a qualification. The median number of deliveries assisted was higher in Phnom Penh than in the two provinces. Previous experiences of attending obstetrics and midwifery training courses differed by region.

**Table 2:** Characteristics of the participants, by province

Province	Phnom Penh		Kampong Cham		Svay Rieng		Total	
Number of participants	101		255		167		523	
Number of health facilities	17		93		47		157	
Age								
21–25	14	14%	29	11%	34	20%	77	15%
26–30	35	35%	97	38%	79	47%	211	40%
31–35	15	15%	32	13%	19	11%	66	13%
36–40	4	4%	7	3%	2	1%	13	2%
41–45	7	7%	20	8%	2	1%	29	6%
46–50	18	18%	47	18%	14	8%	79	15%
51–55	2	2%	12	5%	11	7%	25	5%
56–60	6	6%	11	4%	6	4%	23	4%
Median (IQR)	31 (27–46)		31 (28–46)		28 (26–33)		30 (27–45)	
Qualification and degree								
Midwife								
Bachelor	11	11%	18	7%	6	4%	35	7%
Secondary	69	68%	143	56%	99	59%	311	59%
Primary	8	8%	69	27%	55	33%	132	25%
Nurse								
Bachelor	8	8%	20	8%	3	2%	31	6%
Secondary	4	4%	2	1%	1	1%	7	1%
Primary	1	1%	3	1%	3	2%	7	1%
Number of deliveries assisted in previous month								
2 or less	19	19%	99	39%	64	38%	182	35%
3–5	26	26%	88	35%	65	39%	179	34%
6–10	32	32%	61	24%	32	19%	125	24%
11 or more	24	24%	7	3%	6	4%	37	7%
Median (IQR)	6 (3–10)		3 (2–6)		3 (2–5)		4 (2–7)	
Previous training experience								
Health Centre Midwife	11	11%	60	24%	32	19%	103	20%
Partograph	33	33%	107	42%	55	33%	195	37%
Basic EmONC	50	50%	46	18%	87	52%	183	35%

### Knowledge on intrapartum care

Table 3a shows that the overall mean score was 58% (SD 19%). Only 3% of the participants scored more than 90%. Table 3b shows knowledge on monitoring items using the partograph. The lowest correct answer rates were for moulding (18%), amniotic fluid (44%), maternal body temperature (41%), and maternal pulse (44%). For knowledge on criteria for diagnosis, only 62% and 49% of the participants correctly answered the questions on ‘commencement of active phase of the first stage of labour’ and ‘diagnosis criteria for post-partum haemorrhage’, respectively. For the knowledge on recommended intervals of monitoring, the correct answer rates were between 33 and 57%. This indicates that more than half of the participants do not know the standard methods of monitoring during labour.

**Table 3:** Knowledge on intrapartum care

a. Overall score in 26 items								
Province	Phnom Penh		Kampong Cham		Svay Rieng		Total	
[n]	[101]		[255]		[167]		[523]	
0–10%	1	1%	0	0%	0	0%	1	0%
11–20%	4	4%	1	0%	10	6%	15	3%
21–30%	7	7%	7	3%	12	7%	26	5%
31–40%	13	13%	19	7%	26	16%	58	11%
41–50%	17	17%	33	13%	26	16%	76	15%
51–60%	17	17%	37	15%	30	18%	84	16%
61–70%	18	18%	58	23%	36	22%	112	21%
71–80%	16	16%	59	23%	19	11%	94	18%
81–90%	5	5%	30	12%	7	4%	42	8%
91–100%	3	3%	11	4%	1	1%	15	3%
Mean (SD)	54.1% (19.3%)		63.2% (17.1%)		51.5% (18.5%)		57.7% (18.7%)	

**b**. Scores by each item									
Province		Phnom Penh		Kampong Cham		Svay Rieng		Total	
	[n]	[101]		[255]		[167]		[523]	
I. Knowledge on monitoring items									
Progress of labour	Cervical dilatation	69	68%	196	77%	106	63%	371	71%
	Uterine contraction	74	73%	207	81%	130	78%	411	79%
	Foetal head descent	58	57%	154	60%	77	46%	289	55%
Foetal conditions	Foetal heart rate	97	96%	250	98%	149	89%	496	95%
	Amniotic fluid	50	50%	130	51%	49	29%	229	44%
	Moulding of foetal head	20	20%	68	27%	4	2%	92	18%
Maternal conditions	Pulse	50	50%	121	47%	57	34%	228	44%
	Blood pressure	69	68%	148	58%	89	53%	306	59%
	Body temperature	41	41%	114	45%	57	34%	212	41%
	Urine volume	42	42%	173	68%	62	37%	277	53%
II. Knowledge on criteria for diagnosis or normal range of the items									
Progress of labour	Commencement of active phase	63	62%	150	59%	110	66%	323	62%
	End of the 1st stage	71	70%	234	92%	116	69%	421	80%
	End of the 2nd stage	77	76%	230	90%	119	71%	426	81%
	End of the 3rd stage	71	70%	199	78%	116	69%	386	74%
	End of the 4th stage	48	48%	164	64%	87	52%	299	57%
Foetal conditions	Upper normal limit of FHR	72	71%	197	77%	129	77%	398	76%
	Lower normal limit of FHR	67	66%	193	76%	119	71%	379	72%
Maternal conditions	Diagnosis of hypertension (correct answer rate)								
	0–20%	5	5%	12	5%	10	6%	27	5%
	21–40%	4	4%	9	4%	8	5%	21	4%
	41–60%	15	15%	41	16%	18	11%	74	14%
	61–80%	27	27%	108	42%	71	43%	206	39%
	81–100%	50	50%	85	33%	60	36%	195	37%
	Median (IQR)	79%	(62–86%)	71%	(62–83%)	73%	(62–83%)	74%	(62–83%)
	Diagnosis of PPH	44	44%	149	58%	62	37%	255	49%
III. Knowledge on recommended interval of monitoring									
Progress of labour	Cervical dilataion, 1st stage	43	43%	137	54%	90	54%	270	52%
	Uterine contraction, 1st stage	26	26%	87	34%	57	34%	170	33%
Foetal conditions	FHR, 1st stage	48	48%	169	66%	79	47%	296	57%
	FHR, 2nd stage	23	23%	132	52%	39	23%	194	37%
Maternal conditions	Pulse rate	35	35%	106	42%	55	33%	196	37%
	Blood pressure	43	43%	113	44%	51	31%	207	40%
	Body temperature	33	33%	115	45%	53	32%	201	38%

### Correlating factors with knowledge on process of delivery

Tables 4 and 5 show the results of univariate and multivariate analyses between the characteristics of the participants and their score on the knowledge test, respectively. Univariate analyses revealed that province, age, qualification, and several previous training experiences had statistically significant associations with the score. However, those who had attended health centre midwife and partograph training courses significantly scored less than those who have not received the trainings. The number of deliveries assisted was not associated with the score.

**Table 4:** Relationship between characteristics of the participants and level of knowledge, univariate analysis

Characteristics		n	Score (mean)	[SD]	*p*-value
Province					
Phnom Penh		101	54.1%	19.3%	< 0.001
Kampong Cham		255	63.2%	17.1%	
Svay Rieng		167	51.5%	18.5%	
Age					
21–30		288	62.4%	17.0%	< 0.001
31–40		79	59.8%	18.4%	
41–50		108	49.3%	17.8%	
51–60		48	45.2%	19.7%	
Qualification					
Midwife	Bachelor	35	65.6%	16.0%	< 0.001
	Secondary	309	60.2%	17.7%	
	Primary	132	48.7%	17.2%	
Nurse	Bachelor	33	70.0%	17.2%	
	Secondary	7	44.0%	25.3%	
	Primary	7	35.2%	17.5%	
Number of deliveries assisted (per month)					
2 or less		182	56.4%	19.7%	0.16
3–5		179	59.9%	17.0%	
6 or more		162	56.8%	19.4%	
Previous training experience					
Health center midwife	Yes	103	53.4%	19.2%	0.009
	No	420	58.8%	18.5%	
Partograph	Yes	195	54.0%	17.4%	< 0.001
	No	328	59.9%	19.2%	
EmONC	Yes	183	58.2%	20.0%	0.68
	No	340	57.5%	18.1%

**Table 5:** Relationship between characteristics of the participants and level of knowledge, multiple linear regression analysis

Factors	ß-coeffient	(95% CI)	*p*-value
Province			
Phnom Penh	2.4	(− 1.9, 6.6)	0.27
Kampong Cham	12.7	(9.4, 16.0)	< 0.001
Svay Rieng	Ref		
Age			
21–30	13.1	(7.5, 18.6)	< 0.001
31–40	9.0	(2.8, 15.1)	0.004
41–50	0.3	(− 5.3, 5.9)	0.91
51–60	Ref		
Qualification			
Bachelor MW / Ns	15.9	(6.0, 25.8)	0.002
Secondary MW	11.2	(2.2, 20.2)	0.015
Primary MW	3.4	(− 5.7, 12.5)	0.46
Secondary / Primary Ns	Ref		
Previous training experience			
HC-MW	1.45	(− 2.5, 5.4)	0.47
Partograph	0.05	(− 3.3, 3.4)	0.98

Multivariate linear regression analysis revealed that ‘Kampong Cham province’, ‘younger age’, and ‘higher qualification’ were significantly associated with a higher score. Previous experience in training courses was not associated with the score.

### Misclassification of monitoring items during delivery

The proportion of wrong answers in the three questions on items to be monitored during labour is shown in Table 6. Sixty-one percent of the participants answered ‘uterine contraction’ as an item of foetal condition. ‘Cervical dilatation’ and ‘foetal head descent’ were categorised as a maternal condition by 42 and 44% of the participants, respectively. ‘Amniotic fluid’ was categorised as progress of labour by 35%. Vital signs of a mother were categorised as progress of labour by between 20 and 29%.

**Table 6:** Misclassification of monitoring items during labour [n = 523]

Category	Item	Proportion of incorrect answer		
		Progress of labour	Foetal condition	Maternalcondition
Progress of labour	Cervical dilatation		12.2%	41.9%
	Uterine contraction		61.4%	26.4%
	Foetal head descent		16.6%	44.4%
Foetal condition	Foetal heart rate	19.5%		26.2%
	Amniotic fluid	34.6%		18.9%
	Moulding of foetal head	2.1%		2.1%
Maternal condition	Pulse	22.9%	7.1%	
	Blood pressure	29.3%	5.7%	
	Temperature	19.9%	5.2%	
	Urine volume	21.8%	12.2%	

## Discussion

This study shows that the level of knowledge necessary to monitor and evaluate the labour process is low among skilled birth attendants in the study area. Younger age and higher levels of qualification were significantly associated with higher knowledge. Those who work in Kampong Cham scored significantly higher than those in the other two regions. Previous training courses did not contribute to the current knowledge level.

The total numbers of deliveries in the study participant facilities in 2017 were 8950, 12,860, and 8509 in Phnom Penh, Kampong Cham, and Svay Rieng, respectively, which accounted for 22, 47, and 61% of all births in each region. These figures indicate that a substantial proportion of deliveries were conducted by trained health staff without a higher level of knowledge on monitoring the progress of delivery. Knowledge ensures quality of care through correct gathering and appropriate interpretation of information from a pregnant woman and foetus as well as translation of knowledge into practice. Sharing information on clinical course and its management plan facilitates practicing team medicine. Referring to the framework for quality maternal and newborn care proposed by Renfrew et al. [22], a lack of basic knowledge to monitor and evaluate the labour course hampers the ‘assessment of progress of labour.’ Therefore, further practices will not be realised, including ‘promotion of normal processes and prevention of complications’ and ‘first-line management of complications.’ Maintaining a sufficient level of knowledge is crucial to providing quality care, which contributes to reduce unnecessary morbidity and mortality both in mothers and babies.

This study has shown that age and qualification affected the level of knowledge. Cambodia experienced genocides during the Pol-Pot regime between 1975 and 1979 following a decade of civil conflict. Severe shortage of health care professionals and schools to provide appropriate training was a main issue in health service provision in Cambodia since the early 1980s. One-year training for ‘primary midwife’ and ‘primary nurse’ was created in 1989 to rapidly increase the number of health personnel, although the quality of the courses was untested and questionable [23]. A systematic review on the determinants of quality midwifery care has suggested that short-term training of nurses or midwives is far from the international standard [24]. However, the primary midwife course in Cambodia was maintained until 2015. Durations of undergraduate training are three and four years for secondary and bachelor midwives, respectively [25, 26]. This history and duration of education could be contributing factors for higher knowledge levels among bachelor and secondary midwives than for nurses and primary midwives.

Undergraduate training was observed to be insufficient, and the competency of health care professionals may be improved by the provision of postgraduate training. However, in this study none of the selected training courses showed significant relationship with level of knowledge. Possible factors underlying this finding are either deficiency in the providers of the training or in the participants. For the provider side, it may be because the quality of the training was poor, or little effort such as supervision was made to maintain knowledge of the participants after the training. Although it is difficult to retrospectively evaluate the quality of the previous training, we have confirmed that the selected courses contained appropriate modules for monitoring of labour and delivery. Trainers for the courses were experienced medical doctors or midwives from NMCHC; therefore, low quality of training was unlikely. Studies on training experience in neonatal resuscitation have shown that knowledge and skills deteriorate in the absence of active continuous education with mentoring [27, 28]. Studies on contributing factors of effective learning have shown that the learning environment in the workplace and supportive supervision are key issues [29, 30]. Knowledgeable and skilled preceptors are required who can facilitate other staff members. However, in our study area the average number of birth attendants is 3.5 per facility (542 eligible persons in 157 facilities), and the number of bachelor holders is limited. Because of the shift work nature of health facilities, there would be little opportunity for birth attendants to share information on and discuss findings of labour courses with their colleagues. It implies that it is difficult to expect to conduct self-learning activities in each facility.

The Midwifery Coordination Alliance Team (MCAT) meeting, which was initiated in 2007 in Cambodia, is a mechanism to provide a link between midwives in health centres and hospitals as well as district staff. Its principal components are supervision and feedback for problem solving for common health centre issues, discussion on referred and complicated cases, and updating knowledge to refresh clinical skills [31–33]. Although a national plan intends to scale up the MCAT meetings to all districts [34], to date activities have been organised only in areas where external financial and technical supports are available. Of the three provinces in this study, currently only Kampong Cham has regular MCAT activity [35]. This may explain the higher knowledge level in Kampong Cham than in the other two provinces, although we have no supporting evidence since there was no comparable study using control groups.

Substantial proportions of misclassification of monitoring items during labour were found in this study: 61% answered uterine contraction as a foetal condition; 44% answered foetal head descent, and 26% answered foetal heart rate as maternal conditions; and 29% answered blood pressure as a progress of labour. The participants might have responded without due consideration and rather with an intention of increasing their score. If this is the case, it is a sign of lack of self-confidence, which is a part of professional wisdom of midwifery [36]. These findings also imply that their manner of comprehension was not well structured. As early as the year 1882, Lusk stated that midwifery practice should be based on physiological and pathological investigations, and as a natural outcome of scientific principles [37]. Science requires classification of a phenomenon to understand its background and nature. The International Confederation of Midwives defines key competencies of midwifery as assessment and care of women during labour that facilitates physiological processes and a safe birth; and basic knowledge to fulfill the competency includes anatomy of maternal pelvis and foetus, mechanisms of labour for different foetal presentations, and physiologic onset and progression of labour [38]. Therefore, the misclassifications of monitoring items during delivery imply a lack of understanding among the study participants of physiology and anatomy in pregnancy, labour, and childbirth.

There are possible limitations in this study. First is that recall bias could influence the association between previous training experiences and the knowledge level. The study participants might not declare or forget their previous attendance in training, since two training courses (Health centre midwifery course, and Partograph, the details are shown in Annex-1) were started in the late 1990s. A second possible limitation is that we have not explored other potential factors which affect knowledge and competency of health care professionals, such as the experience of having refresher training. Further studies are required to confirm facilitating factors for maintaining appropriate knowledge in medical practices.

## Conclusions

Our study found that knowledge is insufficient on management of delivery among skilled birth attendants in Cambodia. Previous experience of a single training course did not influence knowledge levels, but MCAT activities might positively contribute. Case-based learning and clinical simulations with repetitive courses for in-service training are recommended to improve knowledge and skill [39, 40]. However, considering the potential deficit in knowledge of physiology and anatomy for midwifery care, the mere provision of clinical training will not change the situation substantially. Physiology and anatomy are foundations of clinical diagnosis, which aims to distinguish pathological states from normal conditions. Building skills and competencies for managing both normal and complicated deliveries is not realistic without a strong background in knowledge of anatomy and physiology. We speculate that the lack of knowledge in basic science abets unnecessary interventions and over-medicalisation in maternity care in Cambodia [41]. Restructuring both pre- and in-service training is required to overcome the constraint in knowledge. Training courses on delivery care should facilitate understanding the mechanism of labour progress as well as its foundation in physiology and anatomy. Studies have suggested that the introduction of conceptual models and core principles of basic science in clinical training would facilitate interplay of knowledge and experience, which enables the participants to explain ‘*what she is doing and wh*y’ [36, 42, 43]. Further intervention with its evaluation should be attempted to improve the knowledge of intrapartum care among birth attendants and to provide quality maternity services in Cambodia.

## Supplementary Information

**Additional file 1: Annex 1.** Details of training in delivery care involved in this study.

## Data Availability

The datasets generated and used for this study are available from the corresponding author on reasonable request.

## Abbreviations

FIGO: International Federation of Gynecology and Obstetrics
ICM: International Confederation of Midwives
ICN: International Council of Nurses
IINeoC: Project for Improving Continuum of Care with focus on Intrapartum and Neonatal Care
IPA: International Pediatric Association
JICA: Japan International Cooperation Agency
MCAT: Midwifery Coordination Alliance Team
MDGs: Millennium Development Goals
NMCHC: National Maternal and Child Health Centre
SBA: Skilled Birth Attendant
UNFPA: United Nations Population Fund
UNICEF: United Nations Children’s Fund
WHO: World Health Organization
