# Supplementary material for: Knowledge on intrapartum care practices among skilled birth attendants in Cambodia—a cross-sectional study
Source: Reprod Health. 2021 Jun 9;18:115. doi: 10.1186/s12978-021-01166-z (PMC8191061; doi:10.1186/s12978-021-01166-z)
Supplement: Supplementary file 1 — Additional file 1: Annex 1. Details of training in delivery care involved in this study. [file 12978_2021_1166_MOESM1_ESM.docx]

**Annex 1.** Details of training in delivery care involved in this study

| Training course | Description |
| --- | --- |
| Health centre midwifery course (HC-MW) | This four-weeks course was created in 1997 as a refresher course for secondary and primary midwives working in health centres. Main organizer of this course is the National Maternal and Child Health Center (NMCHC) in Cambodia. NMCHC has conducted 42 courses between 1997 and 2017, and 480 midwives participated. ^[[1]](#footnote-1)^  This curriculum was updated by NMCHC, JICA, and WHO and validated by the Ministry of Health in 2017. ^[[2]](#footnote-2)^ |
| Partograph | This single short-course training is mainly organized by NMCHC as well as other organizations. The Ministry of Health Cambodia has adapted the partograph, which has latent and active phases, published in 1994.  The number of total participants in this course is not known. |
| Basic Emergency Obstetric and Neonatal Care (BEmONC) | This course was created in 2010 as a national strategy to increase availability, accessibility, quality  and utilization of EmONC services in Cambodia ^[[3]](#footnote-3)^, and has been continued ^[[4]](#footnote-4)^.  NMCHC and provincial health department with EmONC facilities conduct this course with support from UN organizations and NGOs.  Five weeks training is mainly for secondary midwives working in health centres and referral hospitals. Nineteen courses were organised since 2010 for 380 midwives. |

1. Ministry of Health Cambodia. Evaluation of training courses at National Maternal and Child Health Center. 2007. [↑](#footnote-ref-1)
2. Ministry of Health Cambodia. Midwife Training for Health Center. 2016. [↑](#footnote-ref-2)
3. Ministry of Health Cambodia.Emergency Obstetric and Newborn Care (EmONC) Improvement Plan for the period 2010-2015. [↑](#footnote-ref-3)
4. Ministry of Health Cambodia.Emergency Obstetric and Newborn Care (EmONC) Improvement Plan for the period 2016-2020. [↑](#footnote-ref-4)
